# The interplay of CDK4 and CDK6 in melanoma

**DOI:** 10.18632/oncotarget.26515

**Published:** 2019-02-15

**Authors:** Karoline Kollmann, Coralie Briand, Florian Bellutti, Nikolaus Schicher, Stefan Blunder, Markus Zojer, Christoph Hoeller

**Affiliations:** ^1^ Institute of Pharmacology and Toxicology, Veterinary University of Vienna, Vienna, Austria; ^2^ Department of Dermatology, Division of General Dermatology, Medical University Vienna, Vienna, Austria

**Keywords:** melanoma, CDK4, CDK6, PD0332991, angiogenesis

## Abstract

The cyclin-dependent kinases CDK4 and CDK6 promote progression through the cell cycle, where their functions are considered to be redundant. Recent studies have identified an additional role for CDK6 in the transcriptional regulation of cancer-relevant genes such as VEGF-A and EGR1 in hematopoietic malignancies. We show that the CDK4/6 inhibitor PD0332991 causes a significant decrease in tumor growth in a xenotransplantation mouse model of human melanoma. shRNA knockdown of either CDK4 or CDK6 significantly reduces cell proliferation and impedes their migratory capacity *in vitro*, which translates into a strong inhibition of tumor growth in xenotransplantation experiments. CDK4/6 inhibition results not only in the pronounced reduction of cell proliferation but also in an impaired tumor angiogenesis. CDK6 knockdown in melanoma cell lines impairs VEGF-A expression and reduces the potential stimulation of endothelial cell growth. The knockdown of CDK4 ends in similar results. The effect is caused by changes of CDK6 localization, less CDK6 is detected on the VEGF-A promoter. Bioinformatic analysis of human melanoma patient data verifies the key role of CDK6 in tumor angiogenesis in melanoma. The results highlight the importance of the delicate balance between CDK4 and CDK6 in regulating the cell cycle and transcription.

## INTRODUCTION

CDK4 and CDK6 are known as classic cell cycle kinases by forming complexes with D-type cyclins. These complexes phosphorylate the Retinoblastoma protein (Rb) to regulate transition from G1 to S phase [1] [2]. Ablation of either protein in mice is compatible with life and associated with only minor phenotypes whereas the concomitant ablation leads to early embryonic lethality. CDK4/6 have thus for a long time been viewed as redundant proteins [3]. Only over the last years an additional function for CDK6, but not CDK4, as a transcriptional regulator in a kinase dependent and independent manner has been described [4] [5] [6] [7]. CDK6 has been shown to regulate several genes, including EGR-1, FLT-3 as well as the angiogenic factor VEGF-A. This novel function has been demonstrated to be crucial for promoting haematopoietic malignancies, including AML and ALL.

Multiple aberrations in cell cycle regulatory proteins have been described in melanoma. Most prominent are inhibitory mutations in the CDK4/6-inhibitor p16^INK4a^, which have been identified in the majority of primary melanoma samples and melanoma cell lines. In addition germline mutations in CDK4 have been described in families that suffer from hereditary melanoma. These activating mutations of CDK4 (R24C and R24H), in the p16^INK4a^ binding domain, as well as inhibitory mutations in p16INK4a in the germline lead to a 50-fold increase in the risk of developing melanoma [8] [9] [10] [11]. In addition, somatic mutations in codons 22 and 24 of CDK4 have been identified as well [12]. These mutations have been characterized in detail in cell lines as well as in metastatic melanoma and have been causally related to melanoma development [13] [14]. A subgroup of melanomas that harbour combined overexpression of KIT and CDK4 has been characterized [15].

The CDK4/6 inhibitor, PD0332991, has been FDA approved for breast cancer and is currently undergoing early clinical testing in several solid tumors including melanoma. Palbociclib molecules are highly selective for CDK4/6 and has equivalent CDK4/cyclin D3 and CDK6/cyclin D1 potency [16] [17]. Melanoma cells are sensitive to CDK4/6 inhibitors *in vitro* and *in vivo*. This sensitivity might be dependent on expression levels of CDK4, CDKN2A and RB1 [18] [19] [20].

In this study we identify CDK4 and CDK6 as regulators of cell-proliferation, migration and tumor-angiogenesis in melanoma. Chemical CDK4/6 inhibition decreases tumor growth and reduced angiogenesis, which is mimicked by shRNA mediated knockdown of either protein. We found that the availability of CDK6 for transcriptional control is dictated by the expression level and availability of CDK4. This finding defines the importance of a tight and delicate equilibrium between CDK4 and CDK6 in regulating melanoma progression.

## RESULTS

### Inhibition of CDK4 and -6 affects growth and survival of melanoma cell lines

**Figure 1:**
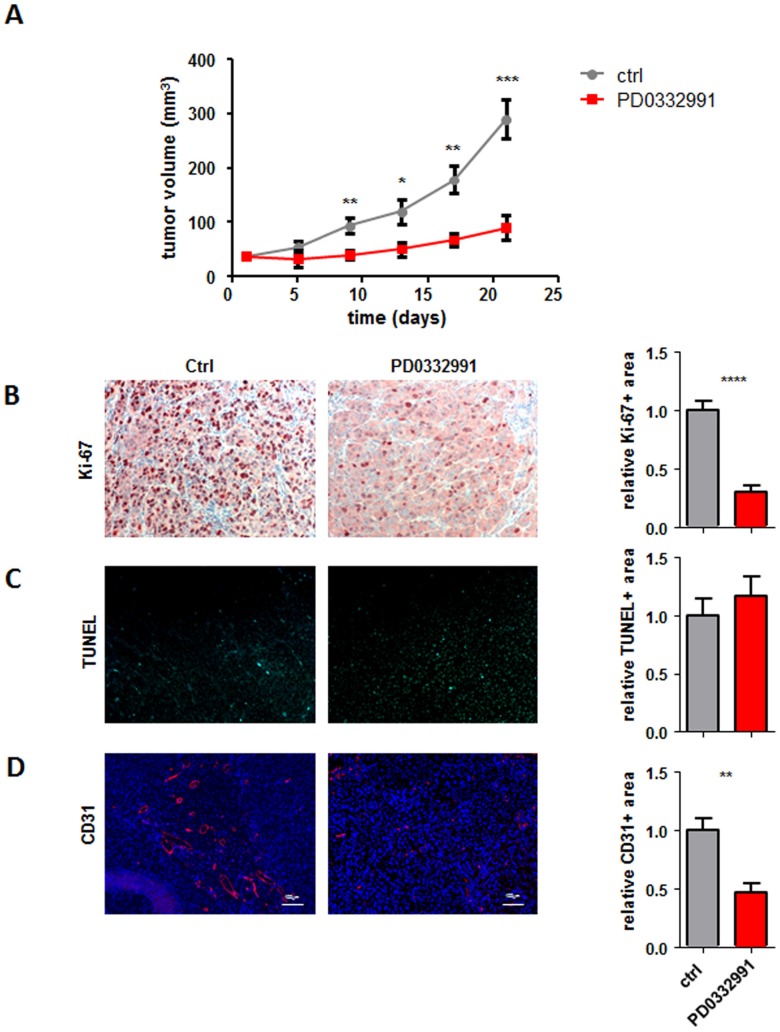
CDK4/6 kinase inhibition reduces subcutaneous tumor formation **A.** 2x106 518A2 melanoma cells were injected subcutaneously into SCID mice. When palpable tumors were present half the mice received daily oral dosing of 150 mg/kg PD0332991. Control animals received solvent on the identical daily schedule. Tumor size was measured every 3 days and tumors were analysed after 21 days (ctrl: *n* = 4; PD0332991: *n* = 8; day9: **; day13: *; day17: **; day21: ***). **B.**-**D.** Immunohistochemical stainings of tumors with and without PD0332991 treatment were analysed for (B) proliferation by Ki-67 (ctrl: *n* = 7; PD0332991: *n* = 5; 4 pictures of each tumor were taken and the average was calculated; Ctrl vs PD0332991, ****), **C.** apoptosis by TUNEL assay (ctrl: *n* = 7; PD0332991: *n* = 7; 4 pictures of each tumor were taken and the average was calculated) and (D) for blood vessels by CD31 (ctrl: *n* = 6; PD0332991: *n* = 5; 5 pictures of each tumor were taken and the average was calculated; Ctrl vs PD0332991, **). Bar graphs depict positive stainings relative to the control. A representative set of pictures is given. Original magnification 20×.

Recent studies showed sensitivity of human melanoma cell lines for CDK4/6 inhibitor treatment [20] [19]. To validate this finding we employed a xenotransplantation model with the human melanoma cell line 518A2 (expressing wildtype CDK4 and p16^INK4a^, data not shown). We also performed dose response curves for this cell line *in vitro* (Supplementary Figure 1). This cell line was xeno-transplanted in SCID mice and the animals subsequently treated orally with either vehicle control or with 150 mg/kg of the CDK4/6 inhibitor PD0332991. As depicted in Figure 1A CDK4/6 inhibitor treatment led to a significant reduction of tumor growth compared to the control group in line with previous *in vitro* studies. Immuno-histochemical stainings of the tumors with the proliferation marker Ki-67 confirmed the pronounced decrease in cell proliferation upon PD0332991 treatment when compared to the control group (Figure 1B). In contrast, we failed to detect any significant changes in apoptosis analysed by terminal deoxynucleotidyl transferase-mediated nick end labelling (TUNEL) staining (Figure 1C).

Melanoma forms highly vascularized tumors [21]. The fact that CDK6 has been implicated in the transcriptional regulation of VEGF-A expression prompted us to analyse tumor angiogenesis. We hypothesized that in addition to growth arrest, the inhibition of CDK4/6 reduces the angiogenic potential of the tumor cells which accounts for the strong inhibition of tumor growth. Stainings and subsequent quantification of blood vessels using the marker CD31 confirmed that concept and verified the pronounced reduction in angiogenesis upon PD0332991 treatment (Figure 1D).

### Knock-down of CDK4 or CDK6 leads to a significant decrease in proliferation/viability

PD0332991 is a highly selective inhibitor of both CDK4 and CDK6 kinase activity. To be able to assign distinct roles to CDK4 and CDK6 for melanoma formation, we performed transient siRNA as well as stable shRNA mediated knockdown of CDK4 and CDK6. Successful siRNA knock down was achieved in 518A2 cells and a second melanoma cell line, LNM1 (Supplementary Figure 2A). The amount of viable cells was assessed by MTS assays after treatment with 10 nM siRNA directed against CDK4 or 6 and showed a reduction in both cell lines after 72 and 96 hours (Supplementary Figure 2B).

**Figure 2:**
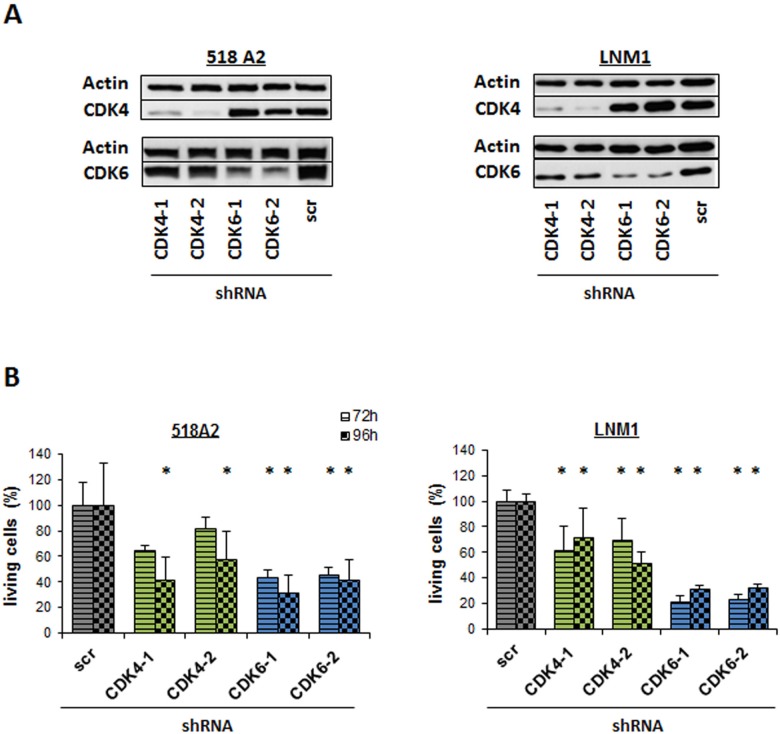
Individual knockdown of CDK4 or CDK6 reduces viability **A.** Western Blot analysis of CDK4 and CDK6 in 518A2 and LNM1 melanoma cells with stable CDK4 or CDK6 shRNA knockdown. 2 different shRNA clones are shown for CDK4 or CDK6. scr = scrambled shRNA. **B.** FACS analysis of living cells 72h and 96h after shRNA knockdown of CDK4 and CDK6 in 518A2 and LNM1 cells (518A2: 72h: shscr *versus*: shRNA CDK6-1, *; shRNA CDK6-2, *; 96h: shscr *versus*: shRNA CDK4-1, *; shRNA CDK4-2, *; shRNA CDK6-1, *; shRNA CDK6-2, *; LNM1: 72h/96h: shscr *versus*: shRNA CDK4-1, *; shRNA CDK4-2, *; shRNA CDK6-1, *; shRNA CDK6-2, *). Experiment has been performed in technical triplicates.

Encouraged by these results we performed stable knockdown of CDK4 or CDK6 using lentiviral transfection of shRNA expressing vectors in 518A2 and LNM1 cells (Figure 2A). In line with the data obtained by transient knockdown we observed a reduced number of 518A2 and LNM1 cells analysed by FACS (Figure 2B). Of note, the effects were more pronounced in cells upon CDK6 knockdown despite the more effective knockdown of CDK4 mRNA irrespective of the cell line. In summary this led us to conclude that both CDKs are important in melanoma, no complete compensation is achieved by the remaining homologue.

### Knock-down of CDK4 or CDK6 results in reduced migration

**Figure 3:**
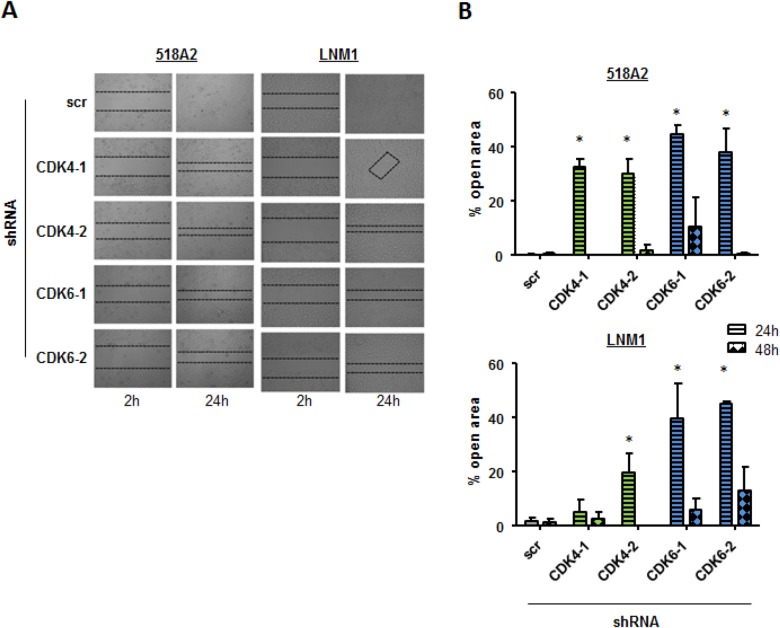
Cell migration is declined in cells with low CDK4 and CDK6 levels A scratch assay was performed to analyze migration of 518A2 or LNM1 cells after shRNA knockdown of CDK4 or CDK6. **A.** After 2h and 24 h, pictures were taken (experiment has been performed in technical duplicates, one representative set of pictures is given) and **B.** migration quantified (% open area after 24/48h relative to 2h; 518A2: 24h: shscr *versus*: shRNA CDK4-1, *; shRNA CDK4-2, *; shRNA CDK6-1, *; shRNA CDK6-2, *; LNM1: 24h: shscr *versus*: shRNA CDK4-2, *; shRNA CDK6-1, *; shRNA CDK6-2, *). 4 pictures per technical duplicate have been analysed.

An important feature of tumor cells is to migrate in order to metastasize. We have recently shown that CDK6 associates with components of the cytoskeleton and regulates cytoskeleton stability. To investigate whether CDK4 or CDK6 are also involved in cell migration in melanoma we performed scratch assays with the 518A2 and LNM1 stable shRNA clones (Figure 3A and 3B). 24 and 48 hours after setting a defined wound in the monolayer of cells the migration of cells with down-regulated CDK4 or CDK6 expression compared to the scrambled (scr) shRNA transduced control cells was significantly impaired. Again, the effects were more pronounced in cells after stable CDK6 knockdown when compared to their counterparts with stable CDK4 knockdown. To rule out any proliferative effects in this assay we performed a scratch assay under Mitomycin C treatment with the same cells. Also in this setting cells with a CDK4 or 6 knockdown migrate slower than the control cells (Supplementary Figure 3).

This finding points at a role for CDK4 and CDK6 in the migratory capacity of melanoma cells.

### Inhibition of CDK4 or CDK6 *in vivo* decreases tumor growth

**Figure 4:**
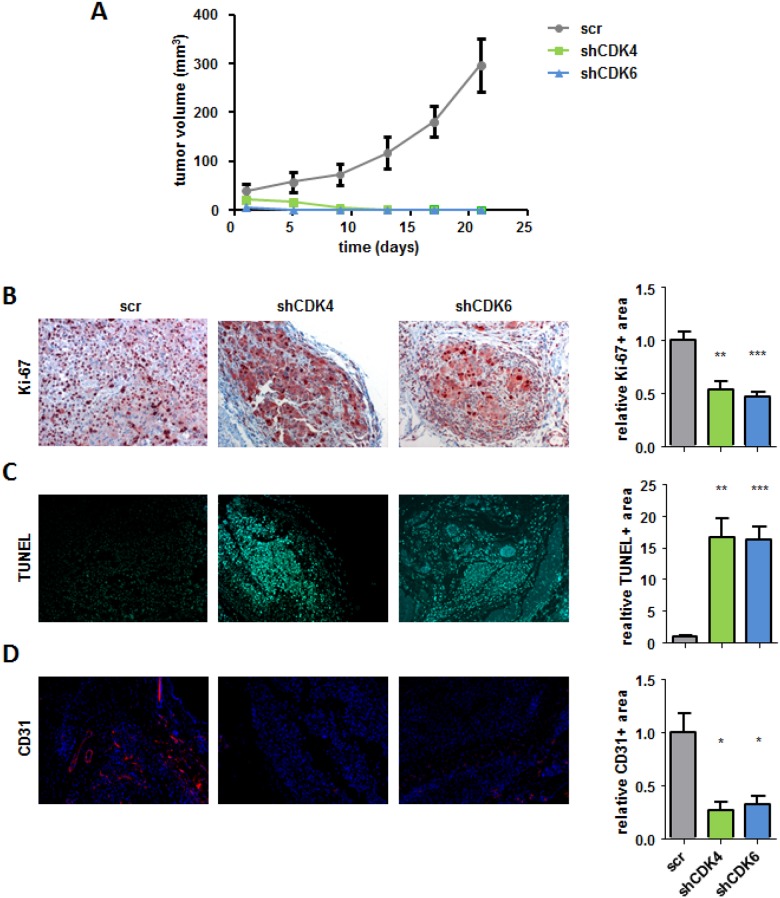
CDK4 and CDK6 shRNA knockdown reduces subcutaneous tumor formation **A.** 2x106 518A2 cells with either CDK4 or CDK6 shRNA knockdown as well as controls were injected subcutaneously into SCID mice. Tumor size was measured every 4 days and analyzed after 21 days (shscr: *n* = 5; shCDK4/6: *n* = 8; day5: shscr *versus*: shCDK6, **; day9: shscr *versus*: shCDK4, **, shCDK6, ***; day13/17/21: shscr *versus*: shCDK4/6, ***). **B.**-**D.** Immunohistochemical stainings of tumors with and without a CDK4 or CDK6 knockdown at day 21 were analysed for the proliferation marker Ki-67 (scr: *n* = 5; shCDK4/CDK6: *n* = 4/5; 4 pictures of each tumor were taken and the average was calculated; scr *versus*: shCDK4, **; shCDK6, ***) **B.**, apoptosis by TUNEL assay (shscr: *n* = 4; shCDK4/CDK6: *n* = 4; 4 pictures of each tumor were taken and the average was calculated; shscr *versus*: shCDK4, **; shCDK6, ***) **C.** and for the blood vessel marker CD31 (shscr: *n* = 4; shCDK4/CDK6: *n* = 3; 4 pictures of each tumor were taken and the average was calculated; shscr *versus*: shCDK4, *; shCDK6, *) (D). A representative set of pictures is given. Original magnification 20×. Bar graphs depict positive stainings relative to the scr control.

To analyse the effects of the single CDK4 or CDK6 knockdown *in vivo* we subcutaneously injected 518A2 shRNA transduced cells into SCID mice (Figure 4A). Irrespective of the shRNA mediated knockdown of CDK4 or CDK6 we failed to observe differences in the initial reaction to tumor cell injection; subcutaneous tumor nodules were readily visible. This could either indicate initial tumor formation or an immune infiltrate as reaction to injection. Importantly tumor nodules completely regressed thereafter and were no longer visible or palpable 9 days after injection of melanoma cells that had either decreased CDK4 or CDK6 expression in contrast to the controls which rapidly increased in volume.

When we analysed the site of tumor cell injection by immune-histochemical stainings 21 days after injection we found residual tumor cells that displayed no signs of proliferation upon stable shRNA knockdown for CDK4 and CDK6 when compared to controls (Figure 4B). TUNEL staining uncovered strong signs of apoptosis upon stable knockdown of CDK4 or CDK6 when compared to control tumors (Figure 4C). Whereas we found pronounced tumor angiogenesis in control tumors, de novo blood vessel formation when analysed by CD31 staining was strongly reduced in the CDK4 and CDK6 knockdown residual tumors. (Figure 4D). In addition we validated the knockdown of CDK4 and 6 in our tumor sections (Supplementary Figure 4). These results indicate that the absence of CDK4 or CDK6 prevents melanoma growth by preventing proliferation and angiogenesis while inducing apoptosis.

### Knockdown of CDK4 or CDK6 reduces VEGF-A secretion/ production

**Figure 5:**
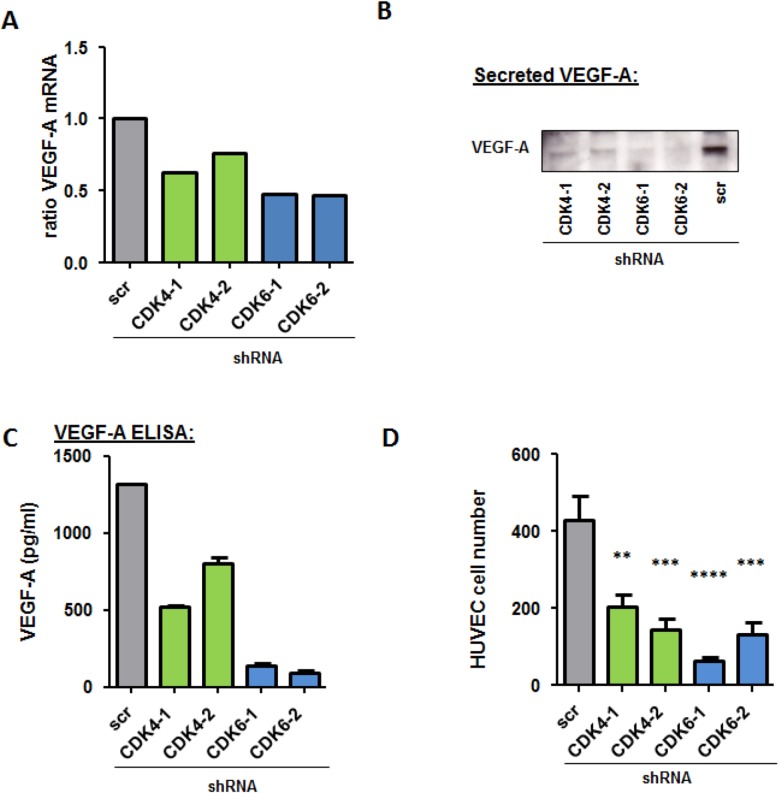
CDK4 and CDK6 reduction decreases VEGF-A production **A.** Relative Vegf-A mRNA levels of 518A2 cells with a CDK4 or CDK6 shRNA knockdown were analyzed by qPCR. The fold change compared to scrambled control is shown. **B.** Western Blot analysis of VEGF-A protein in the supernatant of 518A2 cells with a CDK4 or CDK6 shRNA knockdown. **C.** VEGF-A protein levels (pg/ml) in the supernatant of 518A2 cells with a CDK4 or 6 shRNA knockdown were analyzed with an ELISA experiment. Experiment was performed in duplicates. **D.** Serum free supernatants from 518A2 cells with a CDK4 or 6 shRNA knockdown were collected and added to HUVEC cells. Bar graphs show cell after 72h incubation (shscr *versus*: shCDK4-1, **; shCDK4-2, ***; shCDK6-1, ****; shCDK6-2, ***). Experiment was performed in quadruplicates.

The results so far suggest that pro-angiogenic effects are not only mediated by CDK6 in melanoma but that CDK4 may also be involved. In order to trigger angiogenesis, tumor cells secrete factors in their environment to stimulate endothelial cell proliferation. As it is already known from leukemic cells that CDK6 regulates VEGF-A levels we quantified VEGF-A mRNA levels in the CDK4/6 knockdown cells (Figure 5A). VEGF-A mRNA levels were reduced in all knockdown cells. In addition we detected the secreted VEGF-A in the supernatant of 518A2 cells after 48 hours. In line with the mRNA data this assay demonstrated a clear reduction in both CDK4 as well as CDK6 knockdown cells by western blot (Figure 5B) as well when analysed in an ELISA for VEGF-A protein (Figure 5C). The reduction was more pronounced upon CDK6 knockdown.

In addition, we confirmed a reduction of VEGF-A upon treatment with PD0332991 when we performed an ELISA with treated cells (Supplementary Figure 5A). To determine if this effect is true for more cell lines with different genetic backgrounds we treated a panel of melanoma cell lines with PD0332991 and analysed VEGF-A mRNA levels by qPCR (Supplementary Figure 5B). All cell lines used show a reduction in their VEGF-A mRNA when treated 24h with 300nM of PD0332991. To further test whether CDK4 and CDK6 knockdown reduces secretion of pro-angiogenic factors we examined the effect of supernatant secreted by melanoma cells on the proliferation of human endothelial cells (HUVECs). Endothelial cells survived well for 72h in serum-free medium enriched with conditioned medium from untreated melanoma cells. In contrast, a strong reduction in cell viability of HUVEC cells was observed when the cells were exposed to conditioned media derived from CDK4 or 6 knockdown cells (Figure 5A).

These results suggest that the production and secretion of VEGF-A by melanoma cells is impaired by the stable knockdown of CDK4 and CDK6.

### The availability of CDK6 at the VEGF promoter depends on the presence of CDK4

**Figure 6:**
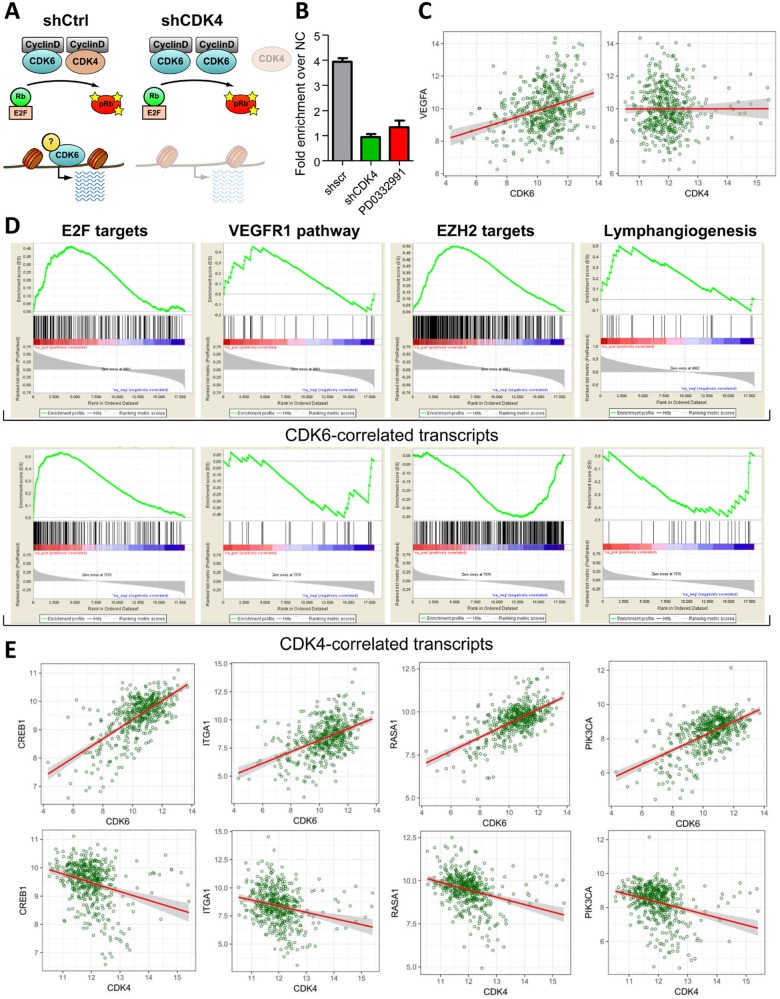
CDK4 levels dictate the transcriptional function of CDK6 **A.** Model of the interplay between CDK4 and CDK6 in regulating cell cycle progression and transcription. **B.** ChIP analysis of CDK6 binding at the VEGF-A promoter in melanoma cells stably expressing a control (shscr) or CDK4-targeting (shCDK4) shRNA or treated with PD0332991 for 24h. Fold enrichment over negative control region is shown. **C.** Scatter plot illustrating the correlation between CDK6 and VEGFA expression levels in melanoma patients. Results are highly significant, details are shown in the Supp. Material and Methods. **D.** Geneset enrichment analysis (GSEA) of regulators of angiogenesis (VEGFR1 pathway, EZH2 targets and Lymphangiogenesis) that were significantly correlated with CDK6 expression in human melanoma patients. The gene expression lists were pre-ranked by CDK6 or CDK4 expression as a control. The E2F target geneset was used as a positive control for the ranked lists. **E.** Scatter plots of representative transcripts involved in tumor angiogenesis that were identified by the GSEA to be significantly positively correlated to CDK6 but not CDK4 expression. Results are highly significant, details are shown in the Supp. Material and Methods.

In summary our data point at a role for CDK4 and CDK6 in controlling angiogenesis in melanoma. So far this function has only been assigned to CDK6. CDK6 but not CDK4 has been found associated with the VEGF-A promoter [4]. We hypothesized that the downregulation of CDK4 alters the availability of CDK6 at the VEGF-A promoter. This includes a concept where the presence and activity of CDK4 as main cell cycle regulator is required to enable CDK6 to fulfill his additional function and to act as transcriptional regulator (Figure 6A).

To test this idea we performed ChIP experiments to analyze the availability of CDK6 at the VEGF-A promoter in the presence and absence of CDK4. In line with our concept we found that CDK6 binding to the VEGF-A promoter was significantly reduced in the absence of CDK4 (Figure 6B) as we have identified CDK6 as one effector molecule for transcriptional regulation of VEGF-A. Cells treated with PD0332991 also show a reduced binding of CDK6 to the VEGF-A promoter.

To study whether our findings in the human cell line relate to patients we analyzed the TCGA dataset that includes microarray data of 331 human cutaneous melanoma cases. In line with our dataset we found a significant correlation between CDK6 and VEGF-A levels that was not observed for CDK4 and VEGF-A expression (Figure 6C). In order to screen for other transcripts that correlate with CDK6 expression we undertook an unbiased approach: We calculated the pairwise spearman correlation coefficients for CDK6 and the other transcripts included in the TCGA microarray study. We created a ranked list of transcripts according to the correlation coefficients and used it to screen for gene sets involved in the regulation of angiogenesis. This approach allowed us to identify gene sets that have been implicated in the regulation of angiogenesis (Figure 6D and 6E, Supplementary Figure 6, Supplementary Table 1). As internal control we tested the link between CDK4/6 and a gene set of E2F target genes. As expected this showed a significant positive correlation for both CDKs and verified the quality of our analysis (Figure 6D). In addition we found clear correlations between CDK6 and the VEGFR1 pathway. Similarly, regulation of lymph angiogenesis was significantly correlated with CDK6 but not with CDK4 expression (Figure 6D, Supplementary Table 2, 3). Another gene set that showed a strong correlation were the EZH2 targets: EZH2 is part of the polycomb repressor complex and is activated by VEGF-A signaling. High levels of EZH2 support tumor angiogenesis by suppressing anti-angiogenic genes and therefore are a predictive factor of poor prognosis [22]. In summary, our data support the unique role of CDK6 as a player regulating angiogenesis whose availability to exert transcriptional functions depends on CDK4.

## DISCUSSION

Although CDK4 and CDK6 have been considered to exert redundant functions in cell cycle regulation [3], there is accumulating evidence that the two proteins have different functions, particularly in cancer [4] [7]. We add a further layer of complexity; both CDK4 and CDK6 are important in melanoma and their crosstalk regulates cell proliferation, viability, migration and angiogenesis. Knockdown of either protein has strong effects that cannot be overcome by the presence of the other.

Prompted by pronounced effects of the CDK4/6 inhibitor palbociclib in melanoma xenograft studies we used specific si/sh- RNA to delete either CDK4 or CDK6 in human melanoma cell lines. Both scenarios resulted in obvious effects on growth and survival of melanoma cells *in vitro* after 72h and 96h. Effects upon CDK6 knockdown seem to be even stronger compared to CDK4 which extended to the migratory behaviour of melanoma cells in a wound healing assay. The concept that the G1 cell cycle kinases CDK4/6 interfere with cell migration and cytoskeletal organisation has already been suggested: recent publications assigned a role to CDK4 in lung cancer cells [23]. The evidence for CDK6 as part of cytoskeletal organisation extends to astrocytes [24] and eryhrocytes [25]. Overexpression of CDK6 in astrocytes is associated with loss of stress fibers and increased motility, in erythrocytes loss of CDK6 results in a decreased cellular stability.

In contrast to PD0332991 treatment which allowed tumor formation in a delayed manner, knockdown of CDK4 or CDK6 resulted in the inability of cells to form any tumor. The initial small palpable nodes completely regressed and were no longer detectable at day 9 and are rather considered an inflammatory infiltrate. At the site of tumor cell injection residual tumor fragments were detectable by immunohistochemistry. In this tumor fragments we found hardly any sign of cell proliferation, but observed clear indications of apoptosis. A reduction in tumor vascularization was observed upon PD0332991 treatment. This discrepancy between kinase inhibitor treatment and protein loss might indicate kinase-independent effects of CDK4/6 on tumor angiogenesis or additional effects on different pathways, e.g. apoptosis. However, VEGF-A has been identified as key factor for tumor angiogenesis and tumor proliferation in malignant melanoma [26] [27]. The effects observed in melanoma were reminiscent of lymphoma models where *Cdk6*^-/-^ lymphoma cells grew significantly slower paralleled by a reduced vascularization which was attributed to reduced VEGF-A expression [2]. In this model VEGF-A expression did not depend on CDK6 kinase activity as it could not be blocked by kinase inhibition. Most importantly reconstitution of a CDK6 kinase-dead mutant in CDK6 deficient cells rescued VEGF-A secretion. This finding first defined CDK6 as transcriptional regulator acting in a kinase-independent manner [4]. In melanoma the situation appears more complex as the treatment of melanoma cells *in vivo* with PD0332991 also impacted on tumor angiogenesis. We can therefore formally not exclude any kinase-dependent effects on VEGF-A in melanoma. We are also not able to assign effects to a distinct cell type as inhibitor treatment was performed *in vivo*. It is attractive to speculate that CDK4/6 regulates additional pro-angiogenic factors in the tumor microenvironment including macrophages or NK cells.

We were also intrigued by the fact that CDK6 but also CDK4 interfered with tumor angiogenesis. Reduction of VEGF-A expression was observed upon downregulation of CDK6 and upon downregulation of CDK4. We propose a model in which the availability of CDK6 at the VEGF-A promoter is determined by the presence or absence of CDK4. Only when CDK4 is the main factor driving proliferation is there enough CDK6 available to exert transcriptional functions at the VEGF-A promoter. This indicates that if CDK4 is downregulated CDK6 has to drive proliferation and is less available at the DNA to regulate transcription. We recently showed that CDK6 acts in concert with the transcription factor c-JUN at the VEGF-A promoter but that D-type Cyclins are not needed for this regulation. , further detailed studies including Chip-Seq and ChIP-Re-ChIP experiments are required to clarify the role of CDK6-Cyclin complexes for the transcriptional function of CDK6 and if such a complex is not needed for transcriptional regulation but is still there This is of particular interest as D type cyclins have also been implicated in transcriptional control [28]. The most convincing support for our concept stems from the analysis of human data sets derived from melanoma patients. We uncovered a solid link between CDK6 expression levels and angiogenesis which was absent for CDK4. This correlation was also detectable for CDK6 and EZH2, which is part of the polycomb repressor complex. The fact that EZH2 as downstream factor of CDK6 was shown to regulate angiogenesis in melanoma indicates that additional angiogenic factors are involved in the CDK6 mediated angiogenesis program [29] [22]. CDK6 regulates angiogenesis in an at least two-fold manner; by directly binding the VEGF-A promoter and by controlling EZH2. It is tempting to assume that CDK6 regulates EZH2 directly and not in an E2F-dependent manner as E2F target genes display a positive correlation with CDK4 and CDK6. Of interest the bioinformatics analysis also uncovered a positive connection between CDK6 and several lymphangiogenic factors. This predicts a potential role for CDK6 in metastasis in melanoma. This idea is further supported by the fact that metastasis requires cell motility and involves cytoskeletal activity. Both potential mechanisms are influenced by CDK4/6. In line with these data a recent study shows that the metastasis of triple negative breast cancer is inhibited by PD0332991 treatment in a mouse xenograft model [30].

In melanoma, activating mutations have so far only been described for CDK4 and are found in melanoma-prone families. The CDK4R24C mutation abolishes the interaction with the inhibitor p16INK4a and renders CDK4 highly active. Our data predict that CDK4R24C dominates the regulation of proliferation and frees CDK6 for transcriptional control. In conclusion, our data support a role for CDK4 and CDK6 as promising therapeutic targets in human melanoma. The effects of PD0332991 on melanoma go far beyond cell proliferation. We postulate a links to metastasis, where migration and angiogenesis are key mechanisms and could be targeted by CDK4/6 inhibitors. Compounds that not only block kinase activity but also interfere with kinase independent functions of CDK6 may be of even greater therapeutic value than the currently available kinase inhibitors.

## MATERIALS AND METHODS

### Cell lines, culture conditions and chemicals

518A2 (courtesy of Dr Peter Schrier, Leiden, the Netherlands) and LNM1 (courtesy of Pr. Petzelbauer, Medical University of Vienna) melanoma cells were cultured in DMEM + GlutaMAX (Gibco Life technologies, Carlsbad, CA) supplemented with 10% heat-inactivated FBS (Sigma-Aldrich, St. Louis, MO). Puromycine Dihydrochloride (Sigma) (2 μg/ml) was added to the medium for the selection of stable transfected shRNA clones. Human umbilical vein endothelial cells (HUVEC courtesy of P. Petzelbauer, Medical University of Vienna) were prepared from umbilical cords by incubation with a 5% collagenase solution, plated in 1% gelatine-coated 75 cm^2^ flask and cultured in IMDM (Lonza Life Technologies) containing 10% Human serum (Gibco), 1% Penicillin- Streptomycin (Gibco), 1% L-glutamine (Gibco), EC growth supplement with heparin (50 μg/ml; Promocell). The cells were cultured at 37°C in a 5% CO_2_ humidified incubator and used between passages three and five after thawing.

The specific CDK4/6 inhibitor PD0332991 used in this study was provided by Pfizer (New York City, NY) and was resolved in DMSO for the *in vitro* studies and in sterile water for the *in vivo* experiments.

### Generation of stable shRNA expression

For stable silencing of CDK4 or CDK6, 3000 cells pro well were plated in 96 well plates in complete medium and incubated with the same amount of MISSION^®^ lentiviral Transduction Particles from Sigma Aldrich according to the manufacturer's instructions. ShRNA clones obtained from the transfection contain the sequence-verified shRNA into the lentiviral plasmids (pLKO.1-puro) with the puromycin selection marker. DMEM complete medium supplemented with 2 μg/mL puromycin (Puromycine Dihydrochloride Ready Made Solution, Sigma) was used for clonal selection of transfected cells. Individual clones were tested for CDK4 or CDK6 expression by western blotting.

### Flow cytometry

Samples were analyzed by a FACSCantoII flow cytometer using FACSDiva software (Becton-Dickinson).

### Western blotting

Cells were lysed in a buffer containing: 1% NP-40, 0,1% SDS, 100 mmol/L NaCl, 50 mmol/L Tris (pH 7,4-7,7), 10 mmol/L EDTA complemented with 10 mmol/L p-nitrophenylphosphate, 250 units/mL aprotinin, 40 μg/mL leupeptin, 1 mmol/L phenylmethylsulfonyl fluoride, 10 mmol/L NaF, and 40 mmol/L β-glycerophosphate. Micro BCA Protein Assay kit (Pierce Biotechnology, Rockford, IL) was used to determine protein concentration. Samples containing equal amounts of protein (20 μg) from lysates of the cultured cells were electrophoresed on a SDS-polyacrylamide gel and transferred to a nitrocellulose membrane. Ponceau red stain and an antibody directed against β-actin were used as loading controls. The nitrocellulose membrane was blocked in DPBS blocking buffer containing 5% BSA and 0,1% Tween 20 for 1h at room temperature and incubated over night at 4°C with the appropriate antibodies. Anti-Rb antibody was obtained from Cell Signaling Technology (Danvers, MA), Anti- Cycline D1, anti-Cycline D3, anti-p16Ink4, anti-CDK4, anti-CDK6 and anti-VEGF-A antibodies from Santa Cruz Biotechnology (Santa Cruz, CA). Following incubation with horse radish peroxidase conjugated second-step antibodies (Santa Cruz Biotechnology) bands were detected using an ECL western blotting detection system (Amersham, GE Healthcare Life Sciences, Buckinghamshire, UK).

### Growth assay (MTS assay)

1500 cells in a final volume of 200 μl per well were plated in 96 well plates. Cells were treated with increasing concentration of PD0332991 (0,1 nM - 300 nM) or solvent control to test the influence of the specific CDK4/6 inhibitor, or treated with 10 nM SiRNA to explore the effect of siRNA mediated down-regulation of CDK4 or CDK6, on growth and viability of the melanoma cell lines. For the stable shRNA transfected cells, 2000 cells were seeded in 200 μl DMEM complete medium per well without any treatment. Cell growth was assessed photometrically using the CellTiter 96^®^ AQueous Non-Radioactive Cell Proliferation Assay (Promega, Madison, WI). Absorbance was measured at 490nM. Proliferation was measured at different time points (after 48 h to 96 h) using a Microplate Reader (Model 680XR, BioRad, Hercules, CA).

### *In vitro* scratch assay

Melanoma cells were seeded on 6-well plates in complete medium. When the cells reached 90% confluence, cell cultures were scratched with a 200 μL sterile pipette tip. Cells were washed with phosphate-buffered saline (PBS) to remove detached cells and complete medium was added with or without PD0332991 (10 nM). Images of the scratch area were captured with a digital camera (Olympus E450, Olympus Imaging America, USA) through an inverted microscope immediately after scratching (T0) and at 24 and 48 hours. To quantify the migratory abilities, the wound area was measured using ImageJ software (nih.gov, Bethesda, MD) and normalized to T0.

### Assay with mitomycin

1,5uM Mitomycin C was added to the media. After 2h and 48 h, pictures were taken

### SCID mouse/human-melanoma xenotransplantation model

All experiments were performed under approval of the university's animal care and use committee. 7-8 weeks old female, pathogen free C.B 17-Scid mice (Harlan-Winckelmann, Germany) were injected subcutaneously into the left flank with 2×10^6^ melanoma cells in 100 μL PBS. Each experimental group consisted of 8 animals each. Experimental groups were

- PD0332991 *vs* vehicle control: 518A2 cell line was used for these experiments. Treatment was started when palpable tumors were present and mice received daily oral dosing of 150 mg/kg of the drug (PD0332991) in 100 μL of sterile water. Control animals received solvent on the identical daily schedule. Tumors were removed after 21 days.

- CDK4 or -6 knock down *vs*. non target control: shRNA mediated CDK4 or -6 deficient lines were compared to the respective 518A2 shRNA non target controls, in regard to tumor formation and tumor growth. In a second group tumors were removed before regression to assess tumor vascularity, proliferation and apoptosis.

Tumor size was measured by caliper and tumor volume was estimated by the formula: [(largest diameter in mm) x (smallest diameter in mm)^2^]/2. Harvested tumors were formalin fixed and paraffin sections obtained from were used for immunohistochemistry analysis.

### Immunohistochemistry

4 μm thick sections were deparafinized and incubated with retrivalbuffer (Dako, Glostrup, Denmark) for 10 min in a pressure cooker at 120°C, 1,5 Bar. After cooling slides were incubated in 1% H_2_0_2_ for 10 min and washed twice in PBS for 5 min. After blocking with blocking buffer containing 1% BSA and 0,1% Tween20 for 20 min (for Ki-67) or 10% goat serum and 0,1% Tween20 for 30 min (for CD31), slides were incubated overnight at 4°C with the appropriate antibody. For Ki-67 staining, samples were incubated with mouse anti-human Ki-67 antibody (Dako) and a biotinylated anti-mouse antibody. Staining was done using AEC reagent (Dako). For CD31 (PECAM-1) immunofluorescence staining with rat anti-mouse CD31 antibody (Histonova, Hamburg, Germany) and DAPI (4′,6-Diamidine -2′-phenylindole dihydrochloride) (Roche Applied Science, Penzberg, Germany) for nucleus staining was used. A Terminal deoxynucleotidyl transferase-mediated nick end labeling (TUNEL) assay to assess apoptosis was performed on tumor sections using the *In situ* Cell Death Detection Kit from Roche (Applied Science) according to the manufacturer’ instructions. Positive areas were quantified by digital image analysis (Axiovision, Zeiss, Wezlar, Germany).

### HUVECs assay

Stable 518A2 transfected melanoma cells were seeded at 7 × 10^5^ cells in 25 cm^2^ flasks. After 24h cells were washed twice with PBS and 6 mL serum free medium (Opti-MEM^®^, Life technologies) was added per flask. Supernatants were collected at 24 hours and sterile filtered to eliminate cellular fragments. Next, 2,3 × 10^5^ HUVEC pro well were plated in 6 well plates. After 24 hours, cells were washed twice with PBS and 2 mL per well of the different supernatants were added to the HUVECs. After 72h, four representative pictures per well were captured with a digital camera (Olympus E450, Olympus Imaging America,USA) through an inverted microscope and cells were counted using ImageJ software (nih.gov, Bethesda, MD).

### VEGF-A quantification/ ELISA

A fixed number of 518A2 and LNM1 cells were cultured in serum free medium (Opti-MEM^®^, Life technologies) and treated with PD0332991 (0, 100, 500 and 1000 nM) for 48 hours. Stable CDK6 and CDK4 shRNA clones for 518A2 melanoma cells in comparison to the respective control cells were cultured in OPTIMEM and VEGF-A concentration was measured after 48 hours. VEGF-A protein concentrations in the supernatant of the cell cultures were determined by using a commercial solid-phase sandwich Enzyme-linked Immunosorbent Assay (Human VEGF-A Platinum ELISA kit, eBioscience, San Diego, CA) according to the manufacturer's instructions. Briefly, a specific monoclonal antibody was coated onto a microplate. Standards and samples were added and a polyclonal detection antibody added. The color developed is proportional to the amount of growth factor present and was read spectrophotometrically at 450 nm. The linear range of the assay is between 16 and 1000 pg/ml.

### Chromatin immunoprecipitation analysis

ChIP experiments were performed in accordance to previously described protocols using anti-CDK6 rabbit polyclonal Antibody (Santa Cruz, H96) [4] [6]. For analysis of CDK6-bound regions, co-immunoprecipitated DNA was analyzed by qPCR (EpiTect HRM PCR Kit, Qiagen) for VEGF-A promoter binding using a MyiQ device (Bio-Rad). Background was evaluated *via* a negative control region (Human Negative Control Primer Set 1, Active Motif). Primer sequences are available upon request.

### Statistical analysis

Statistical analysis to determine group differences was done by Student *t*- test, 1 way or 2 ways ANOVA multiple test using GraphPad Prism software (Graphpad, San Diego, CA; http://www.graphpad.com). Data are reported as mean values ± SEM. The p values are considered as follows: ^*^p < 0.05; ^**^p < 0.01; ^***^p < 0.001 and ^***^p < 0.001.

Level 3 normalized Illumina mRNA-Seq data of 469 Skin Cutaneous Melanoma patients from the Cancer Genome Atlas (TCGA) study were downloaded *via* the Broad GDAC Firehose platform (https://gdac.broadinstitute.org/) (Reference). For each of the 17234 annotated transcripts, the Spearman's Rank correlation coefficient to CDK4 and CDK6 was calculated. For the Gene Set Enrichment Analysis (GSEA; http://software.broadinstitute.org/gsea/index.jsp) the indicated gene sets were downloaded from the MSigDB database. A transcript list ranked by the correlation coefficients was analyzed with the standard parameters. All analyses were performed with R 3.2.3. https://www.R-project.org.

### Correlation plots

Correlation between two genes was visualized by drawing a scatterplot of the gene expression (log2 normalized) for both genes for all patient samples using the ggplot2 function of R. The regression line was added by using the geom_smooth subfunction with the method for linear models (lm).

## SUPPLEMENTARY MATERIALS FIGURES AND TABLES


