# Supplementary material for: The interplay of CDK4 and CDK6 in melanoma
Source: Oncotarget. 2019 Feb 15;10(14):1346–59. doi: 10.18632/oncotarget.26515 (PMC6402717; doi:10.18632/oncotarget.26515)
Supplement: Supplementary file 1 [file oncotarget-10-1346-s001.pdf]

The interplay of CDK4 and CDK6 in melanoma

SUPPLEMENTARY MATERIALS

Mutation status of cell lines:

name	BRAF Ex15	NRAS	CDK4	CDK6	CDKN2A Ex1a	CDKN2A Ex 2-1	CDKN2A Ex 2-2
WM3453	WT	Q61K/WT	WT	WT	WT	WT	WT
607B	WT	Q61K/WT	WT	WT	del	del	del
Mel-Juso	WT	Q61L/WT	WT	WT	del	del	del
SK-Mel28	V600E	WT	R24C/WT	WT	WT	WT	WT
518A2	V600E/WT	WT	WT	WT	WT	WT	del
LNM-1	WT	WT	WT	WT	del	del	del

Protein detection by Western Blot:

name	Cyclin D1	Cyclin D3	Rb	p-Rb 780	p-Rb 811
WM3453	3	0	3	2	3
607B	3	0	1	0	1
Mel-Juso	1	0	3	1	3
SK-Mel28	1	0	1	1	2
518A2	3	0	1	1	2
LNM-1	1	0	1	1	1

Silencing by siRNA treatment: For siRNA treatment cells were plated in serum-free medium (OPTIMEM, Gibco, life technologies) at 75000 cells pro well in 6 well plates for western blotting and cell count, and at 1500 cells pro well in 96 well plate for the Cell Proliferation Assay from Promega. After 24 hours to allow them to recover, the cells were incubated with the respective siRNA (two different SiRNA for each CDK4 and CDK6 and one SiRNA negative control) in combination with Oligofectamine® Reagent (Invitrogen Life technologies). Silencer® SelectedValidated siRNA were ordered from Ambion Inc (Austin, TX) and transfection was done according to the manufacturer’s instructions.

PD0332991 (nM)	% cells in sub G1-phase	% cells in G0/G1-phase of living cells	% cells in S-phase of living cells	% cells in G2/M-phase of living cells
0	12,9	62,8	20	18,4
30	14,9	66,6	15,3	19,2
100	18,4	75,5	12,7	12,3
300	18,2	84,4	7,8	8,3
1000	22,1	89	5,1	6,7
3000	22,5	86,1	6,9	8

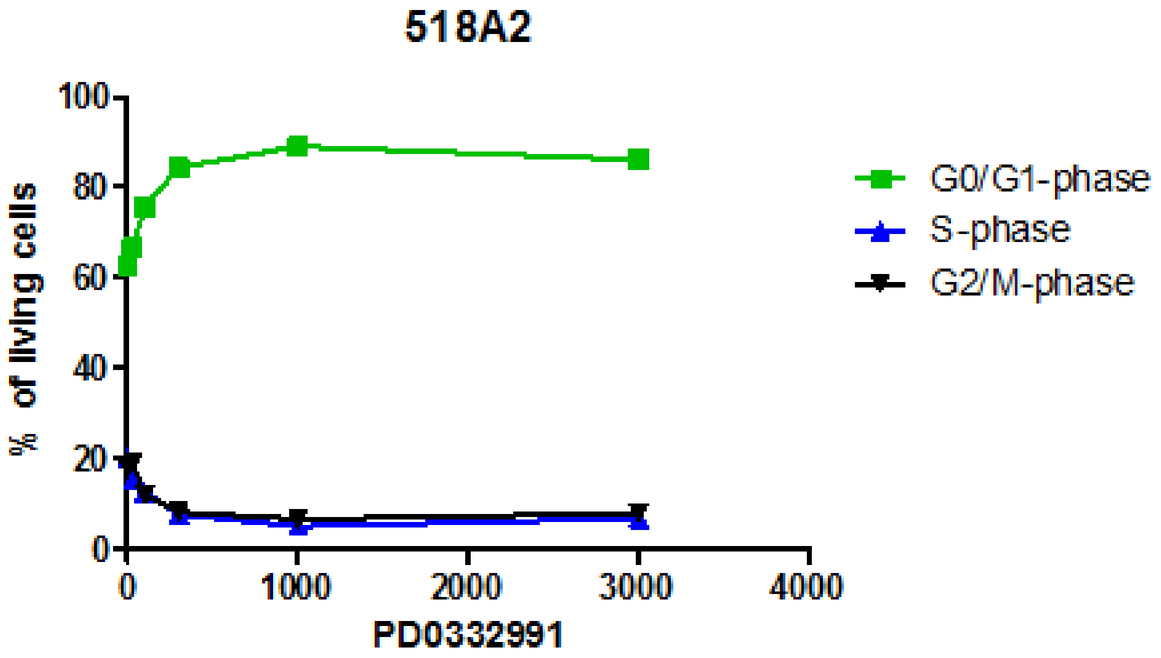

Supplementary Figure 1: Cell cycle profiles of 518A2 cells treated for 24h with 0, 30,100, 300, 1000, 3000 nM PD0332991 were determined by FACS.

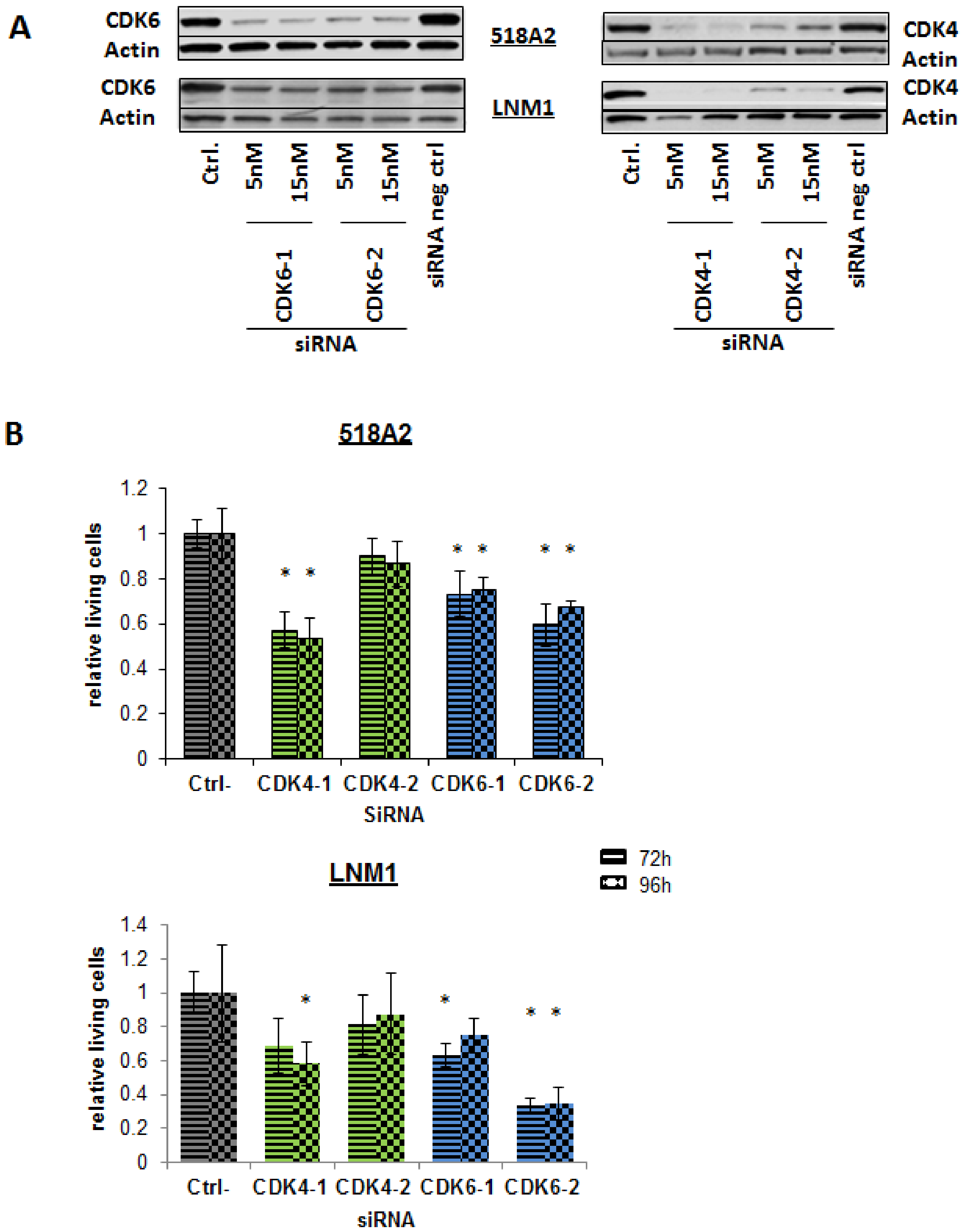

Supplementary Figure 2: A. Western Blot analysis of CDK4 and CDK6 in 518A2 and LNM1 melanoma cells with siRNA CDK4 or CDK6 knockdown. **B.** Analysis of living cells 72h and 96h after siRNA knockdown of CDK4 and CDK6 in 518A2 and LNM1 cells by MTS assay.

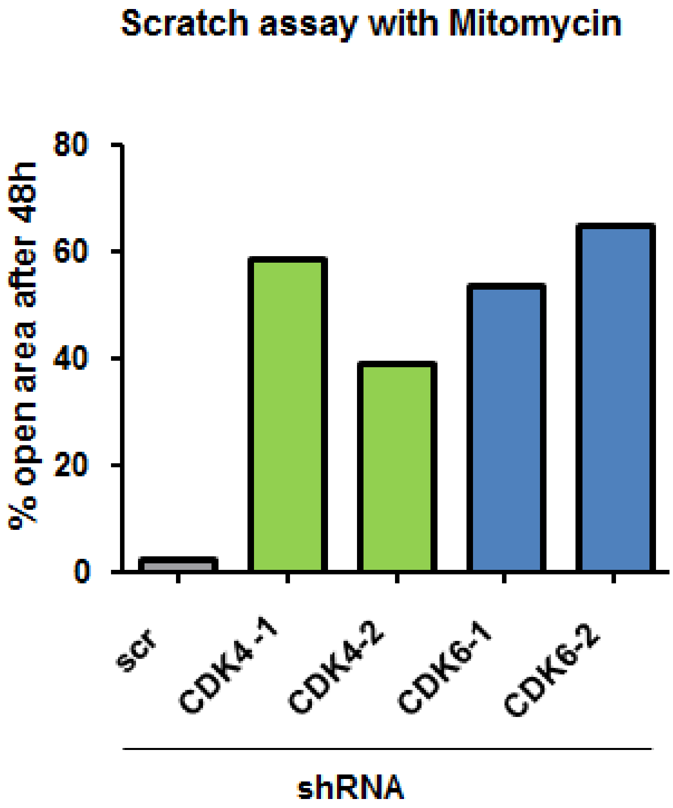

Supplementary Figure 3: A scratch assay was performed to analyze migration of 518A2 cells after shRNA knockdown of CDK4 or CDK6 in combination with 1,5uM Mitomycin C to prevent proliferative effects. After 2h and 48 h, pictures were taken (experiment has been performed in technical duplicates and migration quantified % open area after 48h relative to 2h).

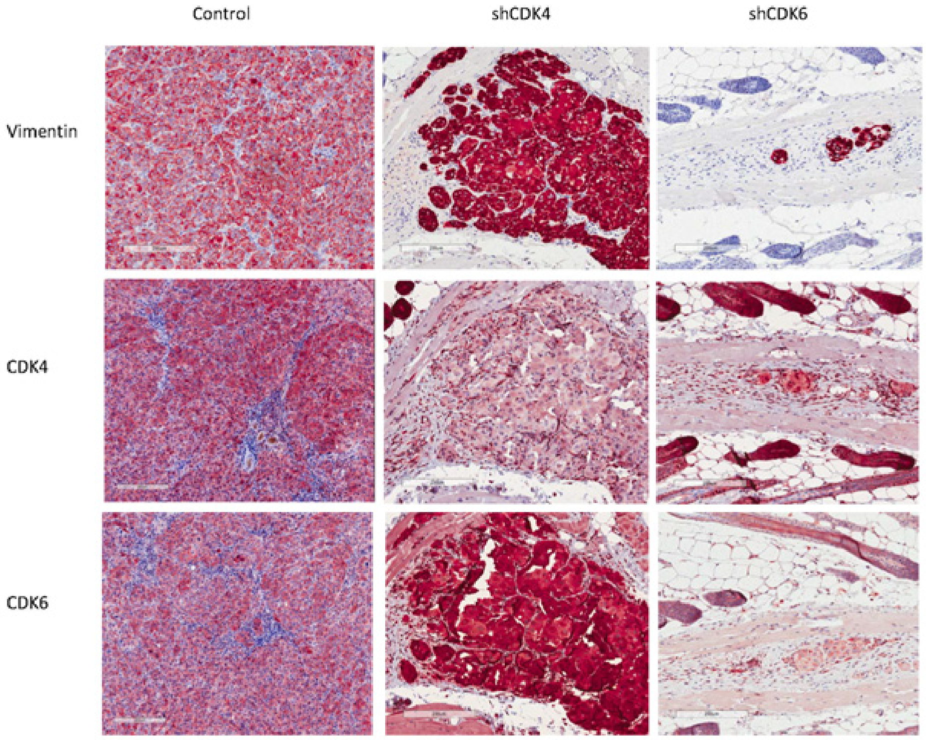

Supplementary Figure 4:´Immunohistochemical stainings of tumors with and without a CDK4 or CDK6 knockdown at day 21 were analysed for CDK4, CDK6 and Vimentin.

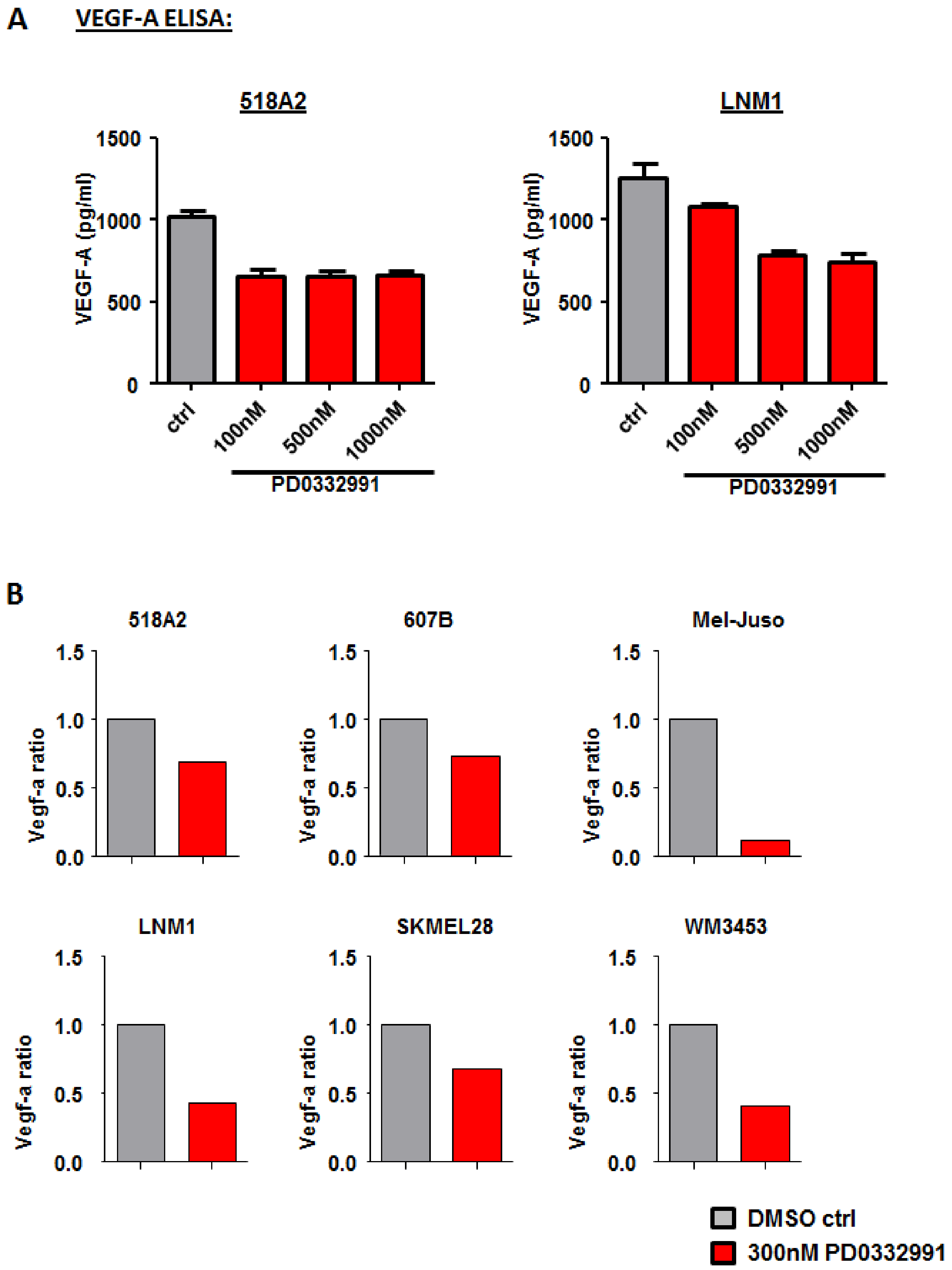

Supplementary Figure 5: A. VEGF-A protein levels (pg/ml) in the supernatant of 518A2 and LNM1 cells treated for 48h with 100, 500 and 1000nM PD0332991 were analyzed with an ELISA experiment. Experiment was performed in duplicates **B.** Relative Vegf-A mRNA levels of a panel of melanoma cell lines (518A2, 607B, JUSO, LNM1, SKMEL28, WM3453) treated for 24h with DMSO, 100nM or 300nM PD0332991 were analyzed by qPCR. The fold change compared to DMSO controls is shown.

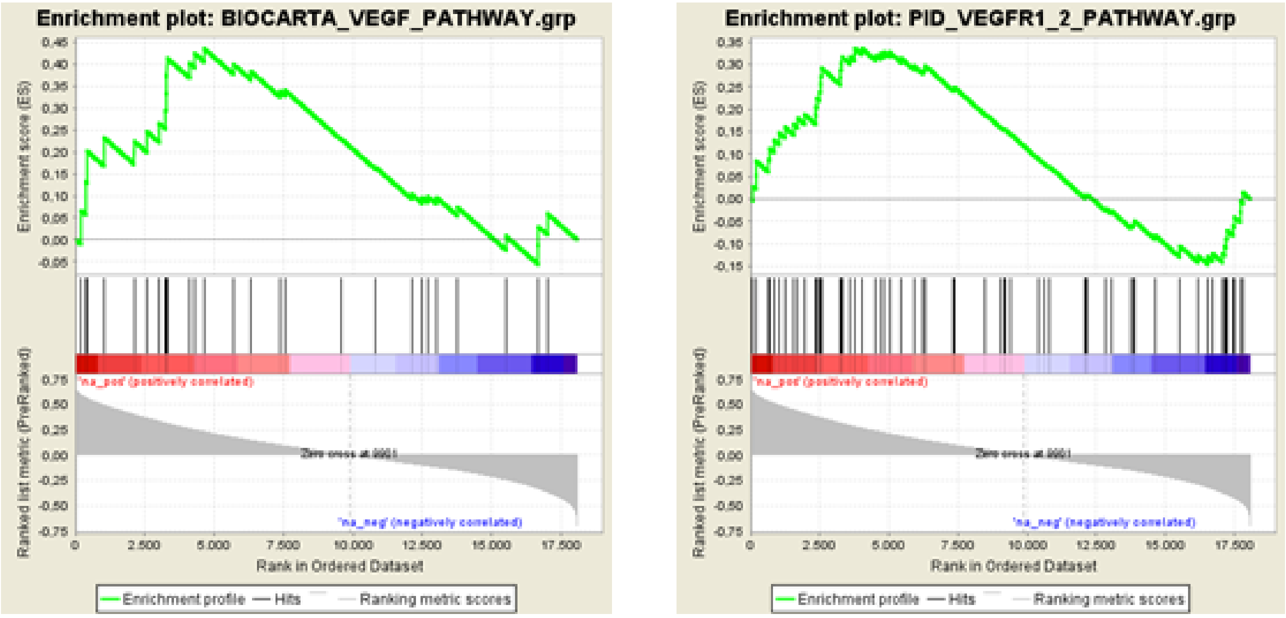

CDK6-correlated transcripts

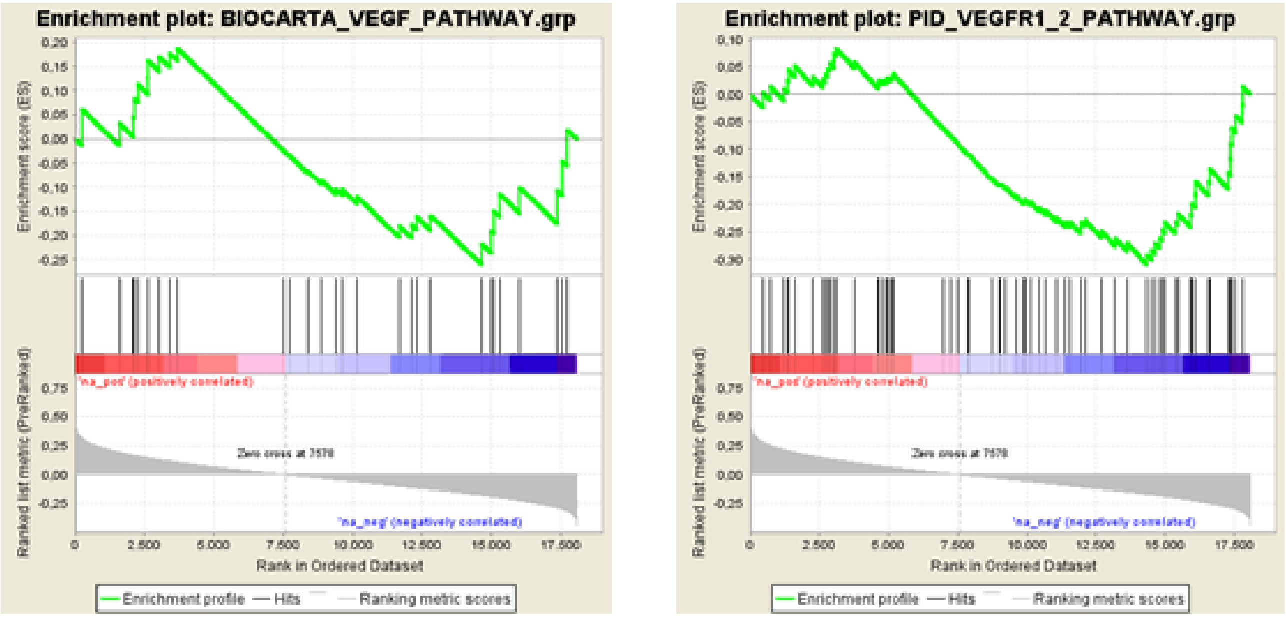

CDK4-correlated transcripts

Supplementary Figure 6: Geneset enrichment analysis (GSEA) of regulators of angiogenesis (VEGF and VEGFR1/2 pathway) that were positively correlated with CDK6 but not CDK4 levels in human melanoma patients.

Supplementary Table 1: Statistical parameters of the Geneset Enrichment Analysis conducted on the pre-ranked lists. The input lists were ranked by CDK4 or CDK6 expression levels.

Dataset	Upregulated in class	GeneSet	Enrichment Score (ES)	Normalized Enrichment Score (NES)	Nominal p-value	FDR q-value	FWER p-Value
Melanoma CDK6	na_pos	BIOCARTA_VEGF_PATHWAY.grp	0.43550268	1.3993926	0.07765668	0.07765668	0.057
	na_pos	PID_VEGFR1_2_PATHWAY.grp	0.33536032	1.2674137	0.117717005	0.117717005	0.099
	na_pos	HALLMARK_E2F_TARGETS.grp	0.41454893	1.783852	0	0	0
	na_pos	PID_VEGFR1_PATHWAY.grp	0.45718548	1.4450976	0.048257373	0.048257373	0.036
	na_pos	LU_EZH2_TARGETS_DN.grp	0.50012964	2.2392774	0	0	0
	na_pos	PID_LYMPH_ANGIOGENESIS_PATHWAY.grp	0.49947712	1.5670592	0.012552301	0.012552301	0.009
Melanoma CDK4	na_neg	BIOCARTA_VEGF_PATHWAY.grp	-0.2606736	-0.88991547	0.6286982	0.6286982	0.425
	na_neg	PID_VEGFR1_2_PATHWAY.grp	-0.3091282	-1.2421473	0.14064516	0.14064516	0.109
	na_pos	HALLMARK_E2F_TARGETS.grp	0.53661275	3.018414	0	0	0
	na_neg	PID_VEGFR1_PATHWAY.grp	-0.4232838	-1.3869885	0.08381503	0.08381503	0.058
	na_neg	LU_EZH2_TARGETS_DN.grp	-0.35409895	-1.7772509	0	0	0
	na_neg	PID_LYMPH_ANGIOGENESIS_PATHWAY.grp	-0.47471014	-1.5586821	0.02130898	0.02130898	0.014

Supplementary Table 2: Summary of the Geneset enrichment results for Lymph angiogenesis. The table shows the rank and the score values of the significantly enriched transcripts.

Lymph angiogenesis pathway

	GENE SYMBOL	RANK IN GENE LIST	RANK METRIC SCORE	RUNNING ES	CORE ENRICHMENT
1	CREB1	7	0.67	0.0921	Yes
2	PIK3CA	195	0.593	0.1635	Yes
3	ITGA2	745	0.52	0.2049	Yes
4	PIK3R1	1016	0.494	0.258	Yes
5	ITGA1	1217	0.474	0.3123	Yes
6	ITGB1	1356	0.463	0.3685	Yes
7	SOS1	1482	0.452	0.4239	Yes
8	ITGA4	1859	0.42	0.461	Yes
9	CRK	2152	0.396	0.4995	Yes

Supplementary Table 3: Summary of the Geneset enrichment results for VEGFR1 pathway. The table shows the rank and the score values of the significantly enriched transcripts.

VEGFR1 pathway

	GENE SYMBOL	RANK IN GENE LIST	RANK METRIC SCORE	RUNNING ES	CORE ENRICHMENT
1	RASA1	91	0.62	0.067	Yes
2	PIK3CA	195	0.593	0.1301	Yes
3	PTPN11	638	0.531	0.1673	Yes
4	CD2AP	911	0.504	0.2108	Yes
5	PIK3R1	1016	0.494	0.2624	Yes
6	NCK1	1237	0.473	0.3051	Yes
7	CBL	2408	0.377	0.2841	Yes
8	HSP90AA1	2483	0.371	0.323	Yes
9	PRKCA	2579	0.363	0.36	Yes
10	FLT1	3236	0.316	0.3603	Yes
11	HIF1A	3258	0.314	0.3957	Yes
12	VEGFA	3299	0.312	0.4297	Yes
13	NRP1	3437	0.302	0.4572	Yes
